# Cerebral microinfarct is emergency consequence of Alzheimer's disease: a new insight into development of neurodegenerative diseases

**DOI:** 10.7150/ijbs.55419

**Published:** 2022-01-24

**Authors:** Xinjie Lin, Yong Fan, Fan Zhang, Yao Lin

**Affiliations:** 1 Provincial University Key Laboratory of Sport and Health Science, College of Life Sciences, Fujian Normal University, Fuzhou, Fujian, China; 2 Central Laboratory, Affiliated Fuzhou First Hospital of Fujian Medical University, Fuzhou, Fujian, China; 3 Affiliated Fuzhou Second Hospital of Xiamen University, Fuzhou, Fujian, China

**Keywords:** glymphatic, microinfarct, cognitive dementia, astrocytes, AQP4

## Abstract

Imbalance of Aβ and tau protein production and clearance are the key factors among many causes of Alzheimer's disease that leading to neurons degeneration and cognitive disorders. As a novel approach, glymphatic system quickly clear metabolic waste (especially Aβ and tau) from cerebral environment, and dysfunction of glymphatic system may relate to occurrence of Alzheimer's disease. Microinfarct is a common histopathologic situation occurring in aging brain and leads to dramatic increase the generation of metabolic by-product after neuronal injury, hindering the operation of glymphatic system and suppress cerebral spinal fluid (CSF) and cerebral interstitial fluid (interstitial fluid, ISF) exchange. Microinfarcts destruct the integrity of microvascular and microstructural tissue, result in Aβ deposition and tau phosphorylation that form neurofibrillary tangles and associated with the cause of Alzheimer's disease. Currently, it has been found that glymphatic system is involved in the pathological process of Alzheimer's disease. Improving the function of glymphatic system after cerebral microinfarcts could be developed as a new approach for Alzheimer's disease prevention and treatment. In this review, we will provide in-depth discussion on functional changes of glymphatic system after cerebral microinfarcts, further reveal pathogenesis of Alzheimer's disease and provide a potentially more effective method for treatment of Alzheimer's disease.

## Introduction

Cerebral microinfarct is a common pathological event in patients with dementia and other cerebrovascular diseases 1, 2. About 87% of 60-70 years old people and almost all 80-90 years old people showed subcortical leukodystrophy. It is estimated that the affected individuals have hundreds of microinfarcts 3, 4. The pathological features of cerebral microinfarcts are consistent with known ischemic infarction 5-7. However, the exact pathophysiological cause of microinfarction has not been fully determined. The main underlying mechanisms include occlusive vascular disease, embolism, hypoperfusion, and destruction of the blood-brain barrier. Cerebral microinfarction can lead to the disruption of measurable structural connections in the brain and is associated with dementia unrelated to the pathology of Alzheimer's disease 8. These findings indicate that cerebral microinfarct is an important mechanistic link between cerebrovascular disease and dementia.

Glymphatic perfusion of the central nervous system (CNS) serves multiple purposes in neurophysiology. It is important for delivery of nutrients, specifically glucose 9, circulation and distribution of apolipoprotein E isoforms produced by the choroid plexus 10, and even astrocytic paracrine signaling with lipid molecules. Glymphatic system has been demonstrated to be responsible for transport of the CSF through the brain parenchyma. CSF moves into brain specifically within periarterial spaces and interchanges with ISF, ISF then drains from brain within perivenous channels. Flow of CSF and ISF within glymphatic system, driven by cerebral arterial pulsatility, has been shown to be critical for the elimination of waste products such as Aβ. The glymphatic proposes water flow through AQP4 in astrocyte end-feet, driven by contractions of the arterial wall, generates a directional, convective flow through the brain from para-arterial to paravenous spaces. AQP4 functions as a bidirectional water channel, which facilitates transfer of water in and out of astrocytes in response to osmotic gradients generated by solute transport. In the aged brain and in the model of Alzheimer's disease murine, glymphatic CSF influx is reduced and the clearance of Aβ is impaired. Further, glymphatic impairment is a prominent feature of cerebrovascular disease, including microinfarction and cerebral ischemia infarction. Following microinfarcts, reduced clearance of cortical interstitial solute was associated with cognitive neurological deficits.

Alzheimer's disease (AD) is associated with the accumulation of toxic aggregates of Aβ and hyperphosphorylated tau in the brain. The concentration-dependent aggregation of Aβ peptides is determined by the balance between production and clearance mechanisms. Aβ peptides are generated from the amyloid precursor protein. Aβ can be cleared from the brain by several mechanisms, including intracellular and extracellular degradation, accumulation in plaques, transcytosis across the blood-brain barrier, and bulk clearance into the CSF. Unlike Aβ, tau is an intracellular protein, hyperphosphorylated tau is thought to be generated in the late stages of neurodegeneration in AD. The spread of tau follows the pattern of neuronal connectivity; therefore, clearance of tau from the brain is much slower than that of Aβ. AQP4-dependent glymphatic pathway for driving the removal of soluble Aβ from the interstitium is an important clearance system. In mice, Aβ is cleared along perivascular, and Aβ clearance was reduced by 55-65% in *Aqp4* knockout mice compared with wild-type mice 9,16. Factors affecting glymphatic ISF bulk flow include molecular size, arterial pulsation, AQP4 expression and localization. In cerebral microinfarcts, long-lasting AQP4 mislocalization and expression decreased from perivascular end-feet to the astrocyte, resulting in reduced perivascular AQP4 availability, which can reduce Aβ clearance.

As a key component of the central lymphatic system, glymphatic system plays an important role in Alzheimer's disease (AD), brain injury (TBI), microinfarcts and other cognitive disorders. Here, we will discuss whether the pathogenesis between neurodegenerative diseases (such as Alzheimer's disease) and cerebral microinfarcts are caused by changes in glymphatic function after microinfarcts. This complex mechanistically glymphatic system could provide new therapeutic targets for neurological disorders associated with immune malfunction and protein aggregation, leading to new therapies.

## Microinfarcts: Invisible lesions

Microinfarcts are a common feature existing among elderly patients, particularly in those suffering mild cognitive dysfunction, vascular or Alzheimer's dementia and are thought to contribute to cognitive decline in elderly patients 11. Cerebral microinfarcts are typically defined as sharply delimited microscopic region of cellular death or tissue necrosis, sometimes with cavitation, which are small ischemic lesions that can be detected by microscope on pathological examination 7. The existence of 1 or 2 microinfarcts found in routine neuropathological specimens signifies the existence of hundreds of microinfarcts in the brain. Microinfarcts may locate in cortical or subcortical regions and are particularly common in patients with vascular cognitive impairment 12. However, they are also frequently found in AD patients and in unselected elderly person. Some community-based research found that the prevalence of microinfarcts was nearly twice higher in person who died with dementia. The appearance of microinfarcts in different brain regions has different effects on cognitive function 13. Thalamic infarctions were tied in with decreased memory performance, while non-thalamic infarcts were tied in with decreased psychomotor speed 14. The status of cognitive deficits associated with microinfarcts has not been thoroughly studied. It is found that microinfarcts are associated with disturbances in episodic memory, semantic memory, and perceptual speed 13. Microinfarcts are best seen under ultrahigh-field MRI at 7T, but it can occasionally be detected on conventional 3T MRI scans. MRI is more sensitive in detecting acute small infarcts detected on diffusion-weighted imaging. A recent study suggested that such small imaging lesions are indicative of an annual incidence of hundreds of new microinfarcts 15, 16.

In addition to causing local tissue damage, microinfarcts can also lead to neurodegenerative changes in distal brain regions 9-10,17. Secondary neurodegeneration after subcortical microinfarcts is mediated by the degeneration of neuronal fiber tracts that linked the initial vascular damage to the distant gray matter, resulting in focal or extensive white matter loss and cortical thinning 18. The underlying mechanism is still unclear but may involve trans-synaptic effects and inflammatory reactions 19,20. Another potential mechanism is selective loss of the volume of vertebral cell in layers Ⅲ and Ⅴ of the dorsolateral prefrontal cortex in patients with microinfarcts which result in degeneration of cortical neurons in the subcortical structure 21. Changes of cortical morphology on MRI images include a decrease in cortical thickness and in sulcus morphology 22. Growing evidence suggested that the effects of subcortical microinfarcts injury on cognitive function are mediated by subsequent cortical gray matter loss 23.

## Distribution of Cerebral Microinfarcts

Data compiled from studies suggest that microinfarcts occur throughout brain, including cortex and subcortical gray and white matter. All cortical and subcortical areas are found to have microinfarcts by comparing incidence of lesions in cortical and subcortical areas. The microinfarcts are then assessed and provide a more complete description of microinfarcts in gray and white matter. The result of two studies specified that no difference was found in appearance of microinfarcts between cortical and subcortical regions, while another study showed that microinfarcts were more common in subcortical regions. However, whether microinfarcts occur preferentially in cortex or subcortical areas was not discussed in most of the studies. Regarding to specific brain areas, cerebral microinfarcts are most common in parietal and occipital areas. Others are only in cortical gray matter. White et al. 24 found that cortical microinfarcts were predominantly located in basal gray matter, while Okamoto et al. 7,25 described them as predominantly in superficial cortex.

Thus, these results suggest that microinfarcts can occur in all brain regions. However, the occurrence of lesions in different brain regions was not compared specifically, but some studies found microinfarcts were more abundant in cerebral cortex.

## Causes and risk factors of microinfarcts

Various underlying causes led to microinfarcts and were able to co-exist in a patient. Three of the main causes are cerebrovascular disease (e.g., cerebral amyloidosis, arteriolosclerosis), microemboli and hypoperfusion. Researches on neuropathology discovered that cerebral microinfarcts are often accompanied by occurrence of severe cerebral amyloid vascular disease 26-28. Patients with cerebral amyloid vascular disease presented with persistent cerebral cortical microinfarcts on 7T MRI and 3T MRI 29-31. Cerebral microinfarcts have been assessed with regard to early markers of hereditary cerebral amyloid vascular disease in the study of cerebral cortex microinfarcts 32.

Neuropathologic studies further explored the relationship between cerebral microinfarcts and cerebral amyloid angiopathy. A study with brain examination of about 1000 community residents, it was found that cortical microinfarcts were accompanied by cerebral amyloid angiopathy 33. Meanwhile, in a study of 80 postmortem patients, all of the patients had multiple cerebral microinfarcts on routine pathological, cerebral microinfarcts in occipital cortex were associated with local cerebral amyloid vascular pathology, but not in frontal cortex and hippocampus 34.

Besides cerebral amyloidosis, cerebral microinfarcts have been found accompany with other small vascular diseases by MRI. Cerebral microinfarcts co-occur simultaneously with lacunar infarcts and were tied in with higher white matter volume and higher cerebral atrophy 29,35-36. The association between cerebral microinfarcts and lacunar infarcts may also be associated with macrovascular disease, as large cortical infarcts and cerebral microinfarcts were discovered co-occurring on MRI 29,36. Notably, atrial fibrillation is also considered to be an important risk factor for cortical cerebral microinfarcts 37,38. Also, biomarkers of cardiac disease, specifically N-terminal pro-brain natriuretic peptide and high-sensitivity cardiac troponin T, were accompanied by cortical cerebral microinfarcts in patients 38. These correlations between cerebral microinfarcts and macrovascular atherosclerosis and heart disease reflect microemboli as a pathogenic mechanism and even hypoperfusion, which has been recognized as another microinfarct formation mechanism in brain 39,40.

A 3T MRI study indicated that cerebral microinfarcts were not associated with diabetes or hypertension, but were significantly associated with hyperlipidaemia 29. However, microinfarcts were tied in with hypertension in a cohort study of 861 participants from common population 36. These inconsistent results suggest that identification of risk factors for cerebral microinfarcts may be influenced by the setting of the studies. In summary, cause of cerebral microinfarcts are heterogeneous, and are associated with different manifestations of small vessel disease, macrovascular disease, and cardiac disease. In most cases, the presence of microinfarction cannot be considered as a sign of particular cause. However, in patients with certain pathological burdens, cerebral microinfarcts may be regarded as a possible pathological sign. The location of cerebral microinfarcts may be associated with different subtypes of small vessel disease, however, MRI researches are limited by the subcortical lesions and current techniques are not able to detect lesions smaller than 1-2mm. With the development of semi-automatic cerebral infarction detection technology and combination of post-mortem MRI and histopathological examination, it would be possible to provide more reliable data on risk factors and causes of cerebral microinfarcts 41.

## Influences of microinfarcts on brain function

Neuropathologic studies have verified the association between cerebral microinfarcts and dementia and cognitive impairment, but the relationship between cerebral microinfarcts and other concurrent pathologies are complex and might require further investigation 42-47. In a cohort study including 238 patients showed that cerebral microinfarcts were tied in with dementia, of which vascular dementia is the most obvious, as 12 out of 22 patients (55%) with vascular dementia have cerebral microinfarcts 29. In 2 year follow-up, cortical cerebral microinfarcts and acute cerebral microinfarcts both predicted that the cognitive performances of the sample of patients suffering from ischaemic stroke or transient ischaemic attack were worse than that of patients without cerebral microinfarcts 37. 3T MRI showed that the cortical microinfarcts were independently associated with dementia, and cortical microinfarcts were significantly associated with poorer overall cognitive performance and task performance in the areas of executive function, visual memory, and verbal memory 36. Notably, these studies considered confounding effects of vascular damage and other markers of neurodegeneration, such as atrophy.

Altogether, these in-vivo MRI studies support neuropathological findings that cerebral microinfarcts are related to poor cognition and are not affected by other age-associated pathologies. However, it is unclear whether these associations are causal or not. Because cerebral microinfarcts are widely distributed throughout the brain, these lesions might cause enough disruption leading to functional impairment, which has been supported by animal studies 41. Microinfarcts could be a marker for other larvaceous vascular lesions that are even more profound effects than lesions themselves and thus can affect the brain without causing visible focal damage.

## The glymphatic system

### Cerebrovascular and the perivascular space

Cerebral arterial circulation consists of anterior cerebral circulation supplied by internal carotid artery, vertebral artery and posterior cerebral circulation. The anterior circulation consists of the middle cerebral artery and the anterior artery, which are connected to posterior circulation through basilar artery, and the posterior cerebral artery by anterior and posterior communication arteries of the Circle of Willis 48. Cerebral arteries extend into pial arteries running through CSF-containing subarachnoid space and subpial space at cortical surface 49,50. As pial arteries penetrate parenchyma, they transform into penetrating arterioles and form a space around vessels called the Virchow-Robin space. CSF filled up the Virchow-Robin cavity and with a layer of pial cells on both inner wall and outer wall facing surrounding blood vessels. Actually, all arterioles, capillaries, and venules in parenchyma are surrounded by astrocytic ends. The distal foot of these vessels forms the outer wall of perivascular space, similar to the circular tunnel surrounding vasculature. As penetrating arterioles shrink deeper into brain parenchyma, the Virchow-Robin spaces become continuous with basal layer. Thus, the Virchow-Robin space disappears before the level of capillaries, and the pericapillary space only consists of basal layer. Basal layer is a thin layer of extracellular matrix, mainly consisted of laminin, fibronectin, type Ⅳ collagen, which contains heparin sulfate proteogly can and other extracellular matrix components. Endothelial cells, pericytes and astrocytes are separated by basal lamina. Endothelial cells pericytes and astrocytes were separated by basement membrane. These three cell types and two other cellular constituents of "the neurovascular unit", smooth muscle cells and neurons, are closely related to extracellular matrix of basement membrane by adhesion molecules, including integrins and anti-myodystrophins 51,52.

Blood enters post-capillary venules from the cerebral capillaries, where the basement membranes of endothelial cell and astrocytes expands and provide a space around vessels for CSF drainage. Generally, blood from interior of the brain flows into larger central/deep veins and out of the cerebral cortex and subcortical white matter through cortical veins that extend to brain surface 53,54. Cerebral arterial circulation was limited to separating anterior and posterior arteries, the areas drained by central veins and cortical veins showed obvious overlapping. Therefore, in certain regions, cortical vein extends down to ventricular wall and in other areas central vein can include the subcortical region 53. Superficial cortical vein anastomosed with deep vein and entry into the superior sagittal sinus. Cerebral venous blood from superior sagittal sinus and deep veins leaves the brain through sinuses draining into sigmoid sinus and jugular veins.

### Structure and functions of glymphatic system

**Figure 1:**
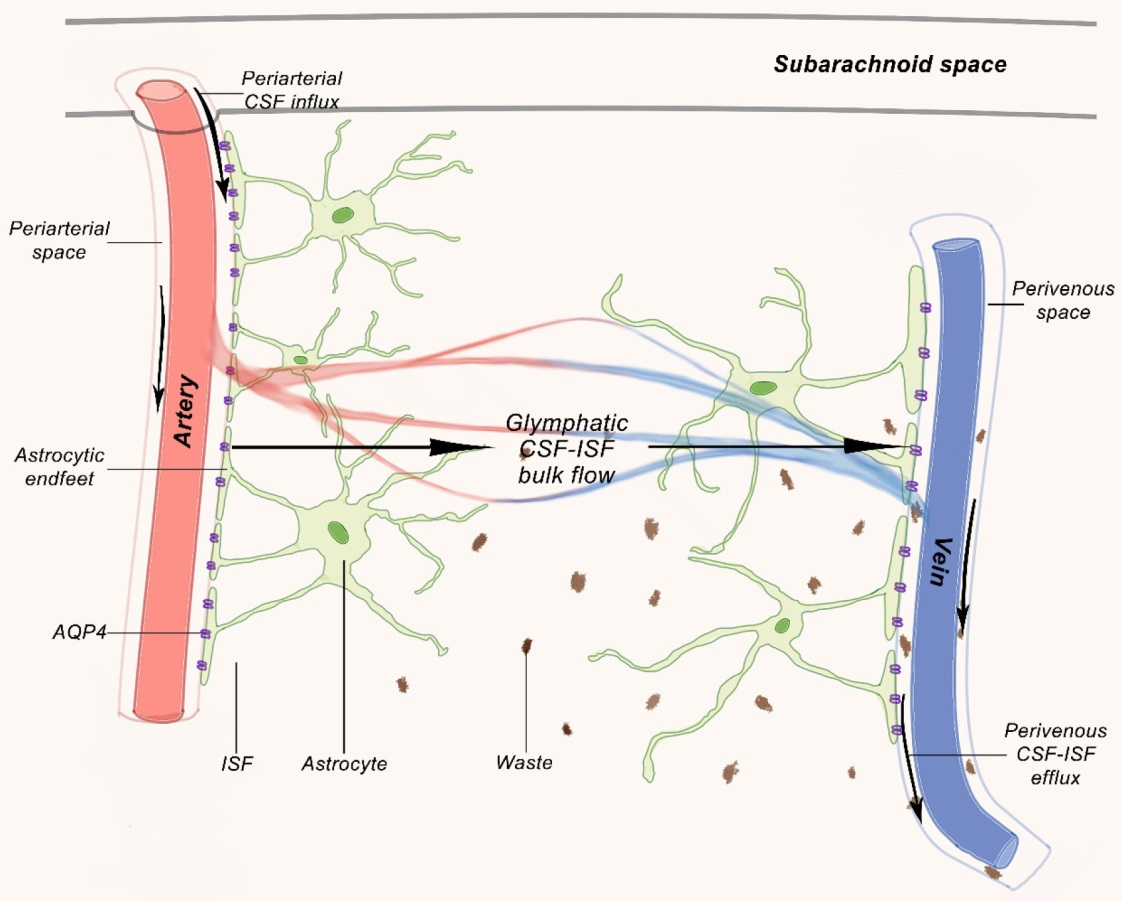
The glymphatic pathway. Studies on rodents demonstrated that CSF from the subarachnoid space flows into the perivascular space of the main cerebral arteries on the brain surface from where it moves along the artery and branches into the venous perivascular space 57.

Recent studies have shown continuous exchange of cerebrospinal fluid and interstitial fluid (ISF). CSF enters the Virchow-Robin spaces from subarachnoid space through a combination of arterial pulse respiration and CSF pressure gradient. The subsequent transport of CSF into dense and complex brain parenchyma is driven by AQP4 water channels. The AQP4 channel is expressed in a highly polarized manner in astrocytic end-feet, which opens up brain vasculature 55,56. CSF enters the parenchyma that prompts the convective interstitial fluid in tissue to flow into the perivenous space surrounding the deep vein. The interstitial fluid is collected in space around veins, where it drains out of the brain and into the neck lymphatic system (Figure 1).

**Figure 2:**
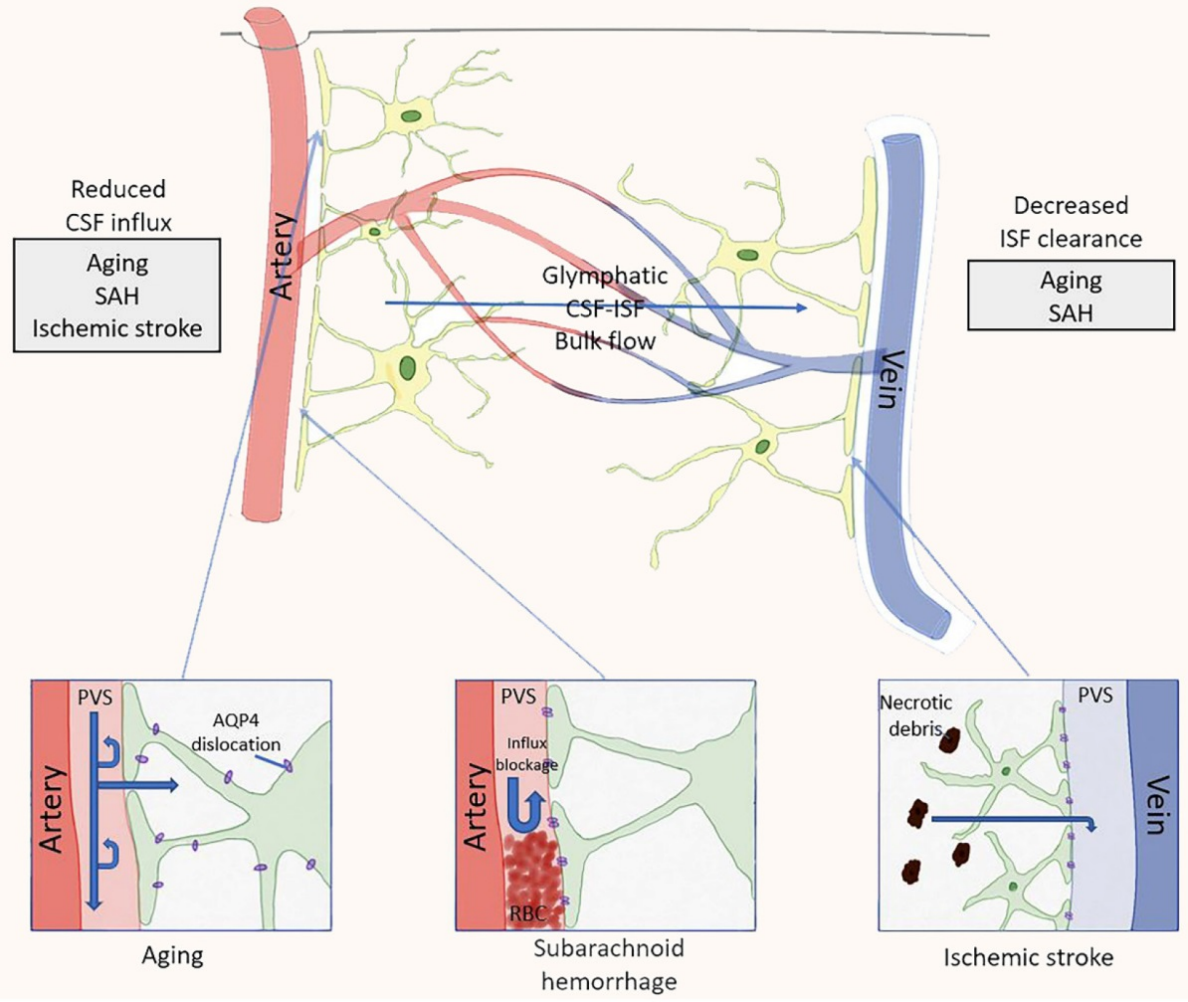
Pathological changes of the glymphatic pathway. In both animals and humans, aging and several diseases have been tied in with a reduction in CSF influx to the lymphatic pathway and/or reduced clearance efficacy. In aging mice, changes in flow are likely caused by decreased vascular compliance, increased expression of AQP4 and dislocation of AQP4 far away from the astrocytic end-feet, all of which led to a decrease in parenchymal influx of CSF. In rodent models of ischemic stroke, necrotic cores are formed within the brain parenchyma, and the surrounding reactive astrocytes form a barrier (glial scar) to contain the injury and the toxic substances formed during necrosis. Contents of the necrotic core leak through the permeable glial scar into the PVS 57.

Two-photon microscopy was applied to characterize the dynamics of the glymphatic system in mice by injecting fluorescent tracer labeled CSF into a large cisterna 55. The experiment showed that the CSF rushes into the brain along cortical pial arteries and flew into Virchow-Robin spaces along penetrating arterioles, then entered parenchyma through periarterial pathway around vascular smooth muscle cells surrounded by peripheral astrocyte endings, where fluorescent tracer rapidly left brain mainly through deep central vein and caudal ventral vein. Periarterial CSF inflow and convective movement through brain parenchyma facilitated clearance of interstitial solutes to perivenous drainage pathway, thereby extending the knowledge of earlier studies with labeled proteins and small molecules 58. This macroscopic clearing mechanism of the interstitial solutes may be particularly important for neurodegenerative diseases, including Alzheimer's disease, which is characterized by accumulation of proteins, including amyloid plaques and Tau tangles (Figure 2). Injection of fluorescent or radio-labeled β1-40 into striatum of mice showed that β1-40 was rapidly cleared from brain along glymphatic paravenous efflux pathway. Furthermore, CSF tracer movement imaging in AQP4 knockout mice showed 65% reduction in parenchyma CSF flux compared to wildtype mice and 55% reduction in striatal clearance of radioactive β-amyloid 55. Thus, the paravascular glymphatic pathway driven by AQP4-dependent bulk flow is the primary clearance pathway for cerebral parenchymal liquid solutes 56.

Therefore, we postulate that central nervous system (CNS) is the only organ system that lacks lymphatic vessels to assist in the removal of interstitial waste. Recent studies have identified the glymphatic system, a glia-dependent perivascular network that assists lymphatic function in the brain 59. Glymphatic system is mainly composed of perivascular spaces (PVS) formed by the foot processes of astrocytes around cerebral arteries and veins, and plays a role in drainage and material exchange between CSF and interstitial fluid (ISF). CSF of subarachnoid space pass through pia mater of PVS into artery of PVS in brain parenchyma, through water channel protein 4 (AQP4, aquaporin 4) of astrocyte, CSF and ISF in parenchymal are mixed, and pass vein PVS in brain parenchyma, enter PVS pia mater vein and reach blood circulation through superior sagittal sinus 60.

In an experiment with Oa-45 tracer injected into the lateral ventricle observed tracer flow from ISF to CSF through glymphatic system, and further transferred to neck lymph nodes and into blood, indicating that extracellular solute of brain cells can be cleared out of the brain through glymphatic system. Amyloid β (Aβ) injected into the brain of mice can be rapidly cleared by glymphatic system 61. Clearance of glymphatic system decreased in elderly mice, and the clearance rate of Aβ decreased. Enhanced MRI showed that glymphatic system could clear tau protein injected into mice brain 62. Therefore, glymphatic system involvement in the clearance of neurotoxic metabolites such as Aβ and tau proteins is important for maintaining intracellular homeostasis and normal brain function.

### Dynamics of glymphatic influx

Entry of CSF through the perivascular space is essential for glymphatic ISF-CSF exchange and clearance function. Glymphatic transportation of CSF along the periarterial space, then the convective flow along the brain parenchyma, and outlet of interstitial fluid (ISF) along the perivenous space to neck lymphatic system is an energy-demanding process driven by a variety of mechanisms. The choroid plexus constantly produces CSF, creating a pressure that indicates the direction of fluid flow through ventricular system to subarachnoid space. What drives CSF into the pial along the perivascular space? Particular to arteries, pulsation produced by the smooth muscle cells generate impulses along the entire length of the pial meninges arteries and penetrate from the cortical surface into the perforating arteries of brain. Dobutamine, an adrenergic agonist, significantly increased pulsatile effect of mice and caused a flood of cerebrospinal fluid into parenchyma. Internal carotid artery ligation inhibited the pulsation in the opposite effect. In addition, reduction of pulse waves reduced the exchange of CSF-ISF 63. This suggests that lymphatic activity is driven, at least in part, explains why perivascular internal flow takes precedence around the pulsing arteries rather than around the cerebral veins.

### Factors affecting glymphatic system

#### a) AQP4

**Figure 3:**
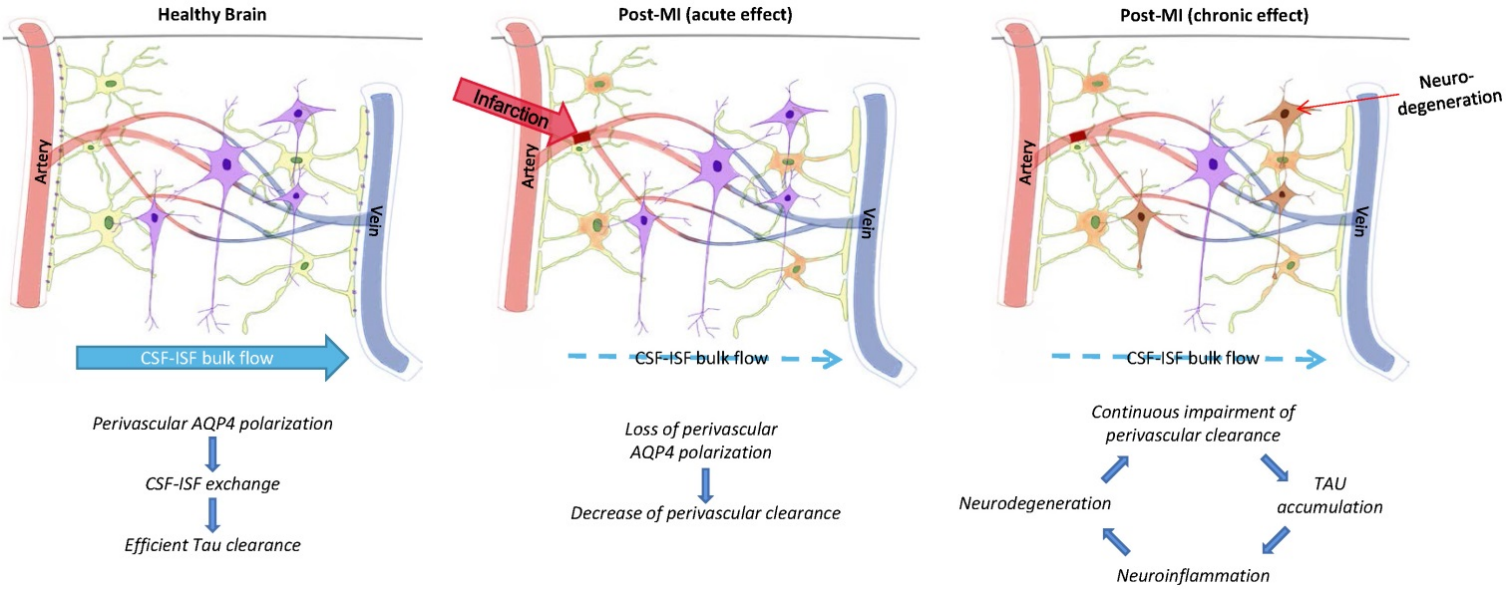
Glymphatic pathway function after infarction promotes tau accumulation. The figure represents the proposed relationship between perivascular AQP4 polarization, glymphatic pathway function, and interstitial tau clearance after microinfarcts. Chronic impact after infarction might be loss of perivascular AQP4 polarization, impairment of paravascular clearance of interstitial tau, promoting tau aggregation, neurodegeneration, and continuous neuroinflammation in the post-ischemia brain 69.

AQP4, which is highly polarized and expressed on the end-feet of astrocytes, acts as a barrier between glymphatic system and brain parenchyma and plays a crucial role in regulating fluid flow between astrocytes, CSF and blood vessels 64. AQP4 mediate glymphatic system to clear Aβ. The deletion of AQP4 promotes the deposition of Aβ, reduce synaptic proteins and brain-derived neurotrophic factors, and aggravate cognitive impairment 65. In addition, the polarization state of AQP4 also affects the material exchange efficiency of CSF and ISF. Highly polarized AQP4 allows ISF to maintain a low-resistance, high-flow output state, thus efficiently removing solutes within ISF (Figure 3). Aging, stroke and craniocerebral injury eliminate AQP4 polarization state, thus affecting the scavenging effect of glymphatic system 66-68. Therefore, highly polarized AQP4, which controls the flow of small molecules of CSF and ISF solutes, is an integral part of glymphatic system.

#### b) Sleep

In glymphatic system, ISF and CSF converge in brain parenchyma. During sleep, the intercellular space of brain cells increased by more than 60%, and the convection exchange between ISF and CSF also increased significantly, which accelerated the clearance of Aβ in CSF and made the concentration of Aβ in CSF during sleep lower than that during awake 69. However, sleep disorder will reduce the internal flow efficiency of PVS, part of AQP-4 polarization will disappear, the ISF outflow resistance will increase and hinder glymphatic system 70.

#### c) Body position

Body position can affect exchange efficiency of CSF and ISF through the change of hemodynamics, thus affecting glymphatic system. It was found that the material exchange efficiency of CSF and ISF was the highest in the right lying position and the lowest in the prone position. The reason may be that the heart position is higher in right lying position, which can promote blood suction and venous return, increase cardiac output, improve cerebral artery pulsation and thus improve the material exchange efficiency of CSF and ISF; while in prone position, the head is relatively higher, CSF outflow along neck vessels increases, which can reduce CSF in the brain, resulting in decreasing of the material exchange efficiency of CSF and ISF 71.

#### d) Cerebral artery pulsation

In the glymphatic system, PVS surrounds cerebral artery, CSF or CSF-ISF mixture flows in PVS, and cerebral artery pulsation can promote rapid movement of solute in PVS in the form of mixing and diffusion, thus completing the material exchange between CSF and ISF 72. Higher pulsation of cerebral artery leads to higher exchange efficiency of CSF and ISF. Meanwhile, elasticity of blood vessel wall or heart rate can change the clearance efficiency of glymphatic system by affecting pulsation of cerebral artery 73.

#### The relationship between dysfunction of glymphatic pathway and cerebral microinfarcts

Microinfarcts destroy the integrity of microvessels or microstructures, reduce exchange of substances between CSF and ISF, promote Aβ and high phosphorylation of tau proteins deposits to form neurofibrillary tangles, and increase the risk of dementia. Dysfunction of glymphatic system leads to inflammatory responses, neurodegeneration, and cognitive impairment 67, 74. Injection of thrombolytic tissue plasminogen activator (tPA) into lateral ventricles of patients with subarachnoid hemorrhage can improve the permeability PVS, material exchange between CSF and ISF and clearance ability of glymphatic system, so as to reduce symptoms of neural defect and neuroinflammatory response 75.

The core of a cerebral microinfarct is filled with microglia and surrounded by reactive astrocytes. AQP4 highly polarized and expressed on astrocytes terminal feet, is the barrier between glymphatic system and brain parenchyma and plays an important role in regulating astrocytes and the flow of fluid between CSF and blood vessels 64. As mentioned above, Aβ in brain can be removed through AQP4 mediated glymphatic system. Clearance of Aβ in brain is related to polar distribution of AQP4 in astrocytes 65. Microinfarcts will lead to loss of polar distribution of AQP4, reducing efficiency of glymphatic system in clearing Aβ and tau, and reducing synaptic protein and brain-derived neurotrophic factor, aggravating cognitive impairment 76. In addition, polarization of AQP4 affects mass exchange efficiency of CSF and ISF, disappearance of polarization of AQP4 increases the outflow resistance of ISF, reduces output of large flow and affects solute clearance in ISF 68.

**Figure 4:**
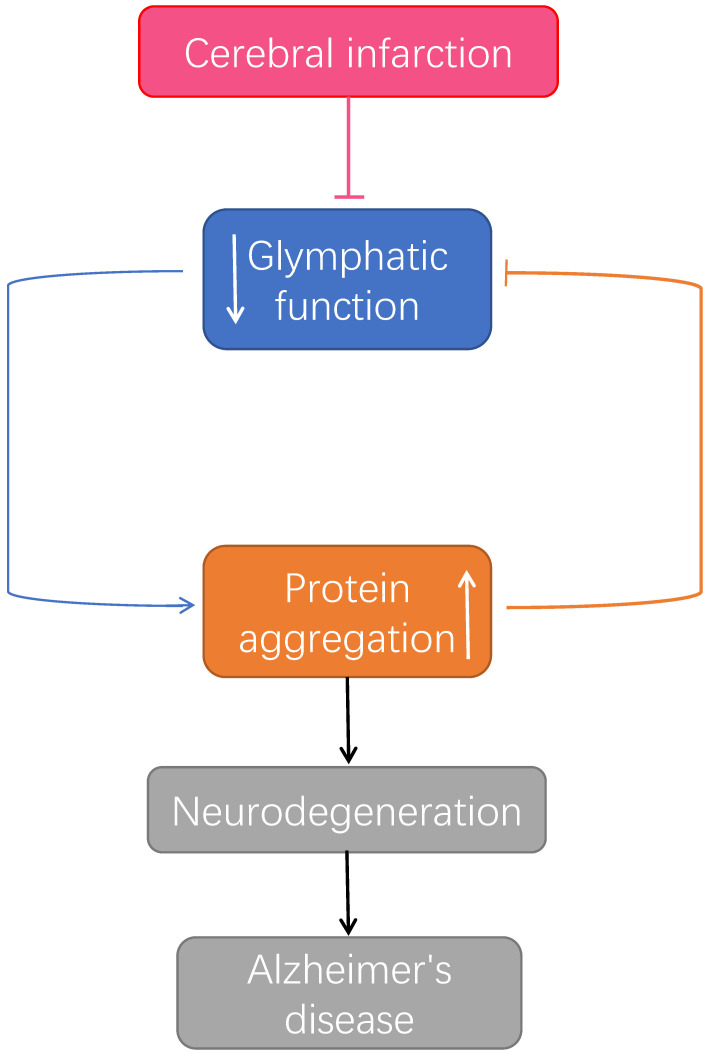
The relationship between microinfarcts, Alzheimer's disease and the glymphatic system. After microinfarcts, malfunctioned glymphatic system accumulates protein aggregation which prompt the developing of neurodegenerative pathology, leading to Alzheimer's disease 83.

It has been reported that deletion of AQP4 gene leads to damaged posttraumatic glial scar formation and affects migration of astrocytes in primary cell culture 77,78. This is because there are two variants of AQP4 expression in astrocytes, which are the result of different transcription start sites: the longer AQP4-M2 variant dominates in healthy brains and exhibits highly polarized perivascular localization 79; the shorter AQP4-M1 subtype is not polarized, but contributes to astrocyte migration 80-82. After occurrence of cerebral microinfarcts, glymphatic system obstruction and Aβ, tau protein aggregation is mainly caused in the expression of two variations of AQP4. Disappearance of AQP4 in perivascular aggregation and polarization state not only affects exchange of CSF and ISF, it also leads to neuroinflammatory response, neurodegeneration and cognitive impairment (Figure 4).

## Conclusions

Microinfarcts are common features in elderly patients, particularly those suffering mild cognitive decline or Alzheimer's disease, and some are thought to play a crucial role in age-related cognitive decline. However, the time course of individual development remains obscure because the microscopic infarcts are typically first identified only upon post-mortem examination. Microinfarcts described in the human clinical are incomplete and diffuse, lesion cores were associated with CD68-positive microglia and GFAP+ astrocyte processes, were typically microscopic (<1mm). GFAP-immunoreactivity and AQP4 polarization within perivascular end-feet, suggesting that water homeostasis and edema clearance may be compromised for a long period of time within a wide region surrounding these diffuse ischemic lesions. AQP4, which is localized to astrocytic end-feet, plays a role in CSF-ISF exchange and interstitial solute clearance through perivascular drainage pathways. The vascular polarization of astrocytic AQP4 is partly lost in reactive astrocytes in diffuse microinfarction brains. The finding that β-amyloid accumulation after microinfarcts was associated with loss of perivascular AQP4 polarization, and that presence of cortical parenchymal AQP4 is associated with CSF-ISF exchange, the microinfarcts related decline in glymphatic function might be in part attributable to dysregulation of astroglial water transport.

There is increasing evidence that reduction of microinfarcts in glymphatic clearance contribute substantially to the accumulation of protein aggregates. Glymphatic system, as a metabolic waste discharge pathway and material delivery system of brain, plays an important role in CSF circulation by promoting the production of CSF and keeping the perivascular space unobstructed which leads to brain homeostasis. Polarizing distribution of AQP4 increase cell gap of brain, improve function of glymphatic system, which is expected to be the target of promoting the clearance of Aβ and tau protein. After microinfarcts, astrocyte activation, AQP4 polarity distribution disappeared, impaired glymphatic pathway function causes a wide range of neural and network damage, the disruption of solute transport surrounding these lesions may similarly drive outsized changes in neuronal network function because of the close association of deep lesions with the underlying white matter. The failure of glymphatic system clearance in microinfarcts might lead to accumulation of β-amyloid and hyperphosphorylated tau proteins and render the brain more vulnerable to developing neurodegenerative pathology or escalate the progression of cognitive dysfunction.

More recent studies have revealed that β-amyloid and fibrillary tangles of tau in Alzheimer's disease are present in the interstitial fluid and CSF. The production and turnover of β-amyloid is strikingly rapid in humans. β-amyloid is not only produced by neurons. in fact, all cells produce β-amyloid. Bulk clearance by the glymphatic system might provide necessary and sufficient removal of extracellular β-amyloid until the end-of-life which time failure in adequate CSF bulk flow leads to accumulation of β-amyloid. This suggests that low activity of the glymphatic system could be a major risk factor for development of neurodegenerative diseases. Cerebral microinfarcts activated astrocytes resulting in loss of AQP4 which might reduce glymphatic CSF influx and the stagnation of CSF influx might accelerate β-amyloid accumulation. In turn, a spiral of protein accumulation, deformation of glymphatic routes and further reduction in protein clearance and pathology.

Glymphatic dysfunction characterized by a failure of interstitial solute clearance is a central feature of aging brain, as well as CNS diseases including Alzheimer's disease, TBI and microinfarcts. While much is known about the regulation of glymphatic pathway, including the roles of cerebral arterial pulsatility, state of consciousness, and head position. There are no glymphatic therapies to intervene in these disease processes at present. The primary assignment of future studies will be identification of a target for regulation CSF-ISF exchange within the glymphatic pathway, to promote improved solute clearance in these diseases.
